# Effect of CAR activation on selected metabolic pathways in normal and hyperlipidemic mouse livers

**DOI:** 10.1186/1471-2164-10-384

**Published:** 2009-08-19

**Authors:** Tadeja Režen, Viola Tamasi, Anita Lövgren-Sandblom, Ingemar Björkhem, Urs A Meyer, Damjana Rozman

**Affiliations:** 1 Center for Functional Genomics and Bio-Chips, Institute of Biochemistry, Faculty of Medicine, University of Ljubljana, Zaloška 4, SI-1000 Ljubljana, Slovenia; 2 Genome Scale Biology, Biozentrum, University of Basel, Klingelbergstrasse 70, CH-4056 Basel, Switzerland; 3 Division of Clinical Chemistry, Karolinska University Hospital at Huddinge, S-14186 Stockholm, Sweden; 4 Current address: Semmelweis University, Faculty of Medicine, Department of Genetics, Cell- and Immunobiology, H-1445 Budapest, Hungary

## Abstract

**Background:**

Detoxification in the liver involves activation of nuclear receptors, such as the constitutive androstane receptor (CAR), which regulate downstream genes of xenobiotic metabolism. Frequently, the metabolism of endobiotics is also modulated, resulting in potentially harmful effects. We therefore used 1,4-Bis [2-(3,5-dichloropyridyloxy)] benzene (TCPOBOP) to study the effect of CAR activation on mouse hepatic transcriptome and lipid metabolome under conditions of diet-induced hyperlipidemia.

**Results:**

Using gene expression profiling with a dedicated microarray, we show that xenobiotic metabolism, PPARα and adipocytokine signaling, and steroid synthesis are the pathways most affected by TCPOBOP in normal and hyperlipidemic mice. TCPOBOP-induced CAR activation prevented the increased hepatic and serum cholesterol caused by feeding mice a diet containing 1% cholesterol. We show that this is due to increased bile acid metabolism and up-regulated removal of LDL, even though TCPOBOP increased cholesterol synthesis under conditions of hyperlipidemia. Up-regulation of cholesterol synthesis was not accompanied by an increase in mature SREBP2 protein. As determined by studies in CAR -/- mice, up-regulation of cholesterol synthesis is however CAR-dependent; and no obvious CAR binding sites were detected in promoters of cholesterogenic genes. TCPOBOP also affected serum glucose and triglyceride levels and other metabolic processes in the liver, irrespective of the diet.

**Conclusion:**

Our data show that CAR activation modulates hepatic metabolism by lowering cholesterol and glucose levels, through effects on PPARα and adiponectin signaling pathways, and by compromising liver adaptations to hyperlipidemia.

## Background

The liver is the central organ of metabolic and energy homeostasis. It regulates levels of endogenous metabolites such as glucose, triglycerides and cholesterol, and detoxifies xenobiotics. These endobiotic and xenobiotic metabolic processes are frequently regulated by the same nuclear receptors. CAR, the constitutive androstane receptor, was initially described as a pure xenosensor of the liver. It activates the detoxification system in the presence of drugs and endogenous molecules, such as bile acids and bilirubin. Therefore, CAR activators are used to treat cholestasis and jaundice in humans and mice [1-4]. TCPOBOP (1,4-Bis [2-(3,5-dichloropyridyloxy)]benzene) and phenobarbital, two representative CAR activators, have strong tumor-promoting effects in mice. They increase hepatocyte proliferation, suppress apoptosis [5,6] and through c-Myc and FoxM1 mediated pathways stimulate the proliferative program leading to liver hyperplasia [7]. CAR activators also increase metabolism of the thyroid hormone and thereby affect metabolic homeostasis [4].

The fact that CAR is not only a xenosensor but also has a role in the endogenous liver metabolism has recently been accepted. For example, long-term treatment with phenobarbital lowered plasma glucose levels in non-insulin-dependent diabetic patients [8]. This has been explained by CAR-dependent repression of mouse liver gluconeogenesis through a mechanism involving HNF4α and FoxO1 [9-12]. Phenobarbital also increases serum triglycerides in patients and mice [13-16]. This was recognized as a CAR-dependent effect where the CAR pathway interacts with PPARα signaling, which is crucial for maintenance of liver lipid homeostasis [16]. A PPARα-CAR inverse regulation has been implicated in inhibiting the β-oxidation of fatty acids, and CAR inverse agonists were proposed as potential drugs for non-alcoholic fatty liver disease (NAFLD) [16,17]. Another side-effect of phenobarbital is an increase in total serum cholesterol in humans and rats [14,18], which results in increased the hepatic cholesterol synthesis by an as-yet undetermined mechanism [12,18]. Most recently, CAR activation in mice was shown to decrease circulating HDL, probably by down-regulating *ApoA-I *gene expression [19].

Despite numerous studies regarding the beneficial or harmful roles of activated CAR, little is known regarding the role of CAR and its cross–talks with metabolic processes in the liver of cholesterol fed mice. This study describes the systemic effect of CAR activation by TCPOBOP on the mouse hepatic transcriptome and lipid metabolome in a state of diet-induced hyperlipidemia.

## Results

### Cholesterol homeostasis

#### Effect of TCPOBOP

**Figure 1:**
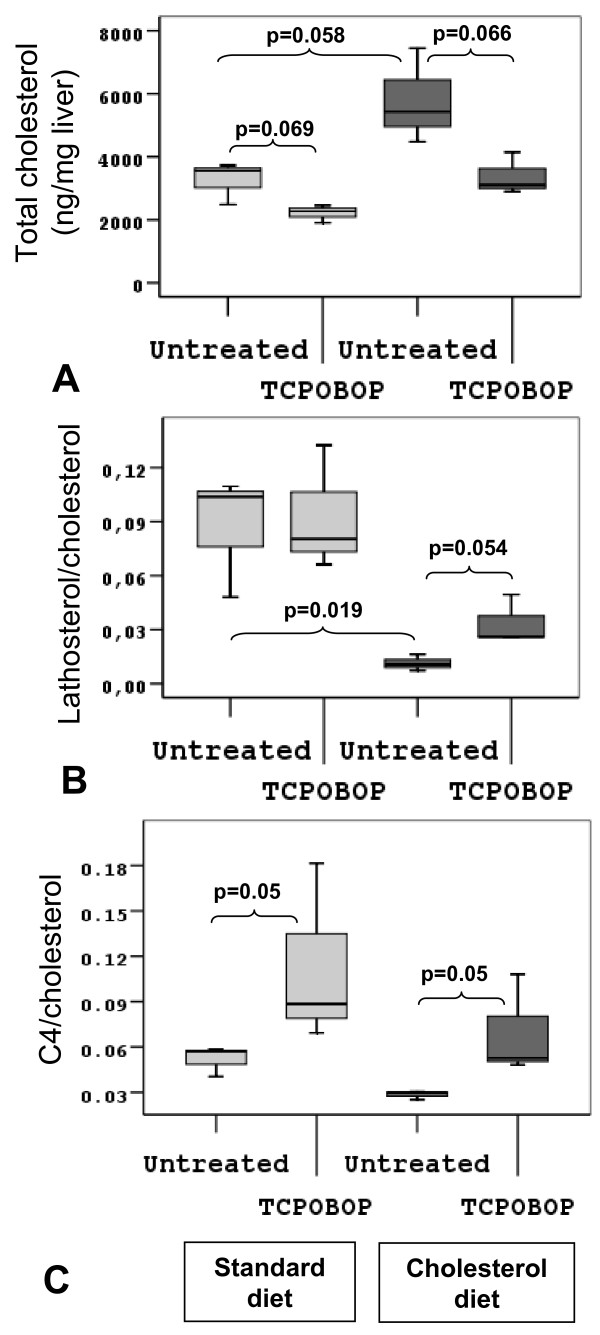
**Liver sterol profiles in TCPOBOP treated mice**. Liver sterol profiles in mice as measured by GC-MS (3 biological samples per group) fed either standard (light box) or 1% cholesterol diet (dark box), and treated either vehicle or TCPOBOP. p-values are indicated between different groups. A. Total liver cholesterol level (ng/mg liver). B. Free lathosterol/total cholesterol level ratio indicates the metabolic rate of liver cholesterol biosynthesis. C. Free C4 (7-α-hydroxy-4-cholestene-3-one)/total cholesterol ratio. C4 is an intermediate in bile acid biosynthesis and shows a metabolic rate of the classical CYP7A1 pathway.

Treatment of normal chow fed mice with TCPOBOP resulted in lower total serum cholesterol, as a result of reduced LDL- and HDL-cholesterol (Table 1). The liver transcriptome data as measured by microarrays (Table 2) provide an explanation. Up-regulation of the LDL receptor gene (*Ldlr*) and the cholesterol ester-break down enzyme (*Lip1*) reflect higher up-take of LDL particles from blood to the liver. At the same time *Acat2*, which is primarily responsible for formation of cholesterol esters destined for VLDL, is down-regulated. Several genes linked to the uptake of HDL to the liver (*Scarb1*), cholesterol efflux to lipid poor apolipoproteins (*Abca1*), or representing parts of either HDL particles or chylomicrons remnants (*Apoa5*, *Apoe*, and *Saa*) were down-regulated. Due to higher liver uptake of LDL cholesterol from serum and lower HDL cholesterol efflux, we would expect an increase in total liver cholesterol. In fact, total liver cholesterol has been lowered (Figure 1A, pale grey boxes). Transcriptome data indicated that this may be due to increased bile acid synthesis, but of the alternative pathways (*Cyp27a1*, *Cyp39a1*, *and Cyp7b1*). The ratio of C4, an intermediate of the classical CYP7A1 pathway, to liver cholesterol (Figure 1C, pale grey boxes), shows an increase in bile acid synthesis, although RNA measurements did not confirm *Cyp7a1 *up-regulation (RT-PCR – Table 3). RT-PCR analysis reveals considerable inter-individual differences in the level of *Cyp7a1 *expression, resulting in a lack of statistical significance. Down-regulation of *Cyp8b1 *and up-regulation of alternative bile acid synthesis could lead to production of more hydrophilic muricholates and therefore to a potential change in composition of the bile [20]. Microarray data also indicate the influence of CAR activation on enterohepatic circulation. As described before, CAR stimulated hepatic export system of bile acids through canalicular (*Abcc2*, *Abcb11*) and basolateral (*Abcc3*) bile acid transporters, but also uptake in the liver (*Slco1a4*) (Table 2). Four genes involved in cholesterol biosynthesis (*Hmgcr*, *Sqle*, *Lss, and Sc5d*) were up-regulated (Table 2 and 3); however, the ratio of lathosterol to cholesterol did not confirm increased cholesterol biosynthesis (Figure 1B, pale grey boxes). Many genes of the SREBP2 signaling pathway, the main regulatory pathway of cholesterol biosynthesis, are up-regulated (*Mbtps1*, *Insig1*, *Insig2*), but not *Srebp2 *itself (Table 3).

**Table 1:** Serum parameters in TCPOBOP treated mice.

**Treatment**	**Vehicle**	**TCPOBOP**	**Vehicle**	**TCPOBOP**
**Diet**	**Standard**	**Standard**	**1% cholesterol**	**1% cholesterol**

Total cholesterol (mmol/L)	3.21 ± 1.52	1.91 ± 0.21*	4.09 ± 0.80*	2.67 ± 0.61^#^
LDL-cholesterol (mmol/L)	0.33 ± 0.05	0.12 ± 0.02*	0.68 ± 0.06*	0.34 ± 0.09^#^
HDL-cholesterol (mmol/L)	1.03 ± 0.05	0.81 ± 0.18*	1.76 ± 0.39*	1.46 ± 0.37
Total triglyceride (mmol/L)	0.54 ± 0.1	0.82 ± 0.12*	1.39 ± 0.43*	1.15 ± 0.25
Glucose (mmol/L)	9.67 ± 0.68	8.59 ± 1.73	12.1 ± 2.16*	10.45 ± 1.83^#^

Serum parameters in mice fed either standard or 1% cholesterol diet and treated either vehicle or TCPOBOP. Each value represents mean ± SD of 6 mice per group. Student t-test was used for calculation of statistical significance (p < 0.05). *Statistical significance compared to vehicle treated group on standard diet. ^#^Statistical significance compared to vehicle treated group on high-cholesterol diet.

**Table 2:** Differentially expressed genes involved in cholesterol homeostasis in mouse liver after TCPOBOP treatment

		**TCPOBOP + normal chow diet**		**Vehicle + cholesterol diet**		**TCPOBOP + cholesterol diet**	
**Gene Symbol**	**Gene Description**	**Log_2 _ratio**	**p-value**	**Log_2 _ratio**	**p-value**	**Log_2 _ratio**	**p-value**

**Cholesterol biosynthesis**							
Sc4mol	Sterol-C4-methyl oxidase-like	nc	nc	-2.50	0.000	1.85	0.001
Cyp51	Cytochrome P450, 51a1	nc	nc	-1.86	0.001	1.27	0.008
Hmgcs1	3-hydroxy-3-methylglutaryl-Coenzyme A synthase 1	nc	nc	-1.79	0.001	1.20	0.015
Fdps	Farnesyl diphosphate synthase 1	nc	nc	-1.73	0.001	0.99	0.026
Fdft1	Farnesyl diphosphate farnesyl transferase 1, Squalene synthase	nc	nc	-1.69	0.000	1.08	0.006
Nsdhl	NAD(P) dependent steroid dehydrogenase-like	nc	nc	-1.35	0.004	0.99	0.033
Lss	Lanosterol synthase	0.60	0.033	-1.31	0.000	0.81	0.012
Mvd	Mevalonate (diphospho) decarboxylase	nc	nc	-1.27	0.002	0.88	0.017
Mvk	Mevalonate kinase	nc	nc	-1.18	0.001	0.86	0.010
Hmgcr	3-hydroxy-3-methylglutaryl-Coenzyme A reductase	0.99	0.001	-1.14	0.001	1.09	0.008
Sqle	Squalene epoxidase	0.49	0.014	-1.11	0.014	nc	nc
Dhcr24	24-dehydrocholesterol reductase	nc	nc	-0.57	0.019	0.54	0.018
Dhcr7	7-dehydrocholesterol reductase	nc	nc	-0.36	0.049	nc	nc
Idi1	Isopentenyl-diphosphate delta isomerase	nc	nc	0.39	0.003	nc	nc
Sc5d	Sterol-C5-desaturase homolog	0.36	0.029	nc	nc	nc	nc
**SREBP signaling pathway**							
Insig1	Insulin induced gene 1	0.82	0.000	nc	nc	nc	nc
Mbtps1	Membrane-bound transcription factor peptidase, site 1	1.72	0.000	nc	nc	2.06	0.003
Insig2	Insulin induced gene 2	1.94	0.000	0.25	0.038	1.24	0.000
**Bile acid synthesis**							
Cyp8b1	Cytochrome P450, 8b1	-2.16	0.000	nc	nc	-0.67	0.036
Cyp7b1	Cytochrome P450, 7b1	0.21	0.014	nc	nc	nc	nc
Cyp27a1	Cytochrome P450, 27a1	0.73	0.000	nc	nc	0.46	0.028
Cyp39a1	Cytochrome P450, 39a1	1.06	0.000	nc	nc	0.91	0.000
**Lipid transport**							
Apoa5	Apolipoprotein A-V	-0.57	0.013	nc	nc	-0.45	0.039
Apoe	Apolipoprotein E	-0.38	0.019	nc	nc	-0.46	0.022
Ldlr	Low density lipoprotein receptor	0.32	0.017	-0.38	0.037	0.42	0.039
Apoa4	Apolipoprotein A-IV	2.23	0.000	1.09	0.000	1.00	0.001
**Transporters**							
Abca1	ATP-binding cassette, sub-family A (ABC1), member 1	-0.39	0.024	0.18	0.040	-0.38	0.015
Abcb11	ATP-binding cassette, sub-family B (MDR/TAP), member 11	0.46	0.001	0.68	0.000	nc	nc
Abcc2	ATP-binding cassette, sub-family C (CFTR/MRP), member 2	0.92	0.000	nc	nc	0.89	0.000
Slco1a4	Solute carrier organic anion transporter family, member 1a4	1.04	0.000	-0.98	0.000	1.66	0.000
Abcc3	ATP-binding cassette, sub-family C (CFTR/MRP), member 3	1.29	0.000	-0.76	0.003	2.06	0.000
Abcg8	ATP-binding cassette, sub-family G (WHITE), member 8	nc	nc	nc	nc	-1.02	0.006
Abcg5	ATP-binding cassette, sub-family G (WHITE), member 5	nc	nc	nc	nc	-0.87	0.008
Slco2b1	Solute carrier organic anion transporter family, member 2b1	-0.40	0.001	0.25	0.009	nc	nc
**Other**							
Scarb1	Scavenger receptor class B, member 1	-0.87	0.000	0.31	0.003	-0.88	0.000
Acat2	Acetyl-Coenzyme A acetyltransferase 2	-0.31	0.010	nc	nc	-0.33	0.019
Lip1	Lysosomal acid lipase 1	0.49	0.000	0.38	0.002	0.28	0.043
Saa2	Serum amyloid A 2	-3.60	0.000	nc	nc	-3.28	0.001
Saa1	Serum amyloid A 1	-3.52	0.000	nc	nc	-3.22	0.001
Saa3	Serum amyloid A 3	-3.39	0.000	nc	nc	-2.87	0.001

Differentially expressed genes involved in cholesterol homeostasis in mouse liver after TCPOBOP treatment or cholesterol diet as detected by the Steroltalk microarray. Data represent log_2 _ratios of TCPOBOP treated versus vehicle treated on the same diet (standard or cholesterol) and vehicle treated group on cholesterol diet versus standard diet. Six animals per group were used and p-value was calculated using t-test as implemented in BRB-Array Tools. GeneBank accession numbers are available in Additional file 1. nc – no change.

**Table 3:** Expression of genes in wild type mice treated with TCPOBOP as detected by RT-PCR

**Treatment**	**TCPOBOP**	**Untreated**	**TCPOBOP**
**Diet**	**Standard diet**	**1%cholesterol *vs *standard diet**	**1%cholesterol diet**

*Hmgcr*	3.09 ± 2.93*	0.27 ± 0.14*	2.42 ± 0.76*
*Cyp51a1*	1.80 ± 1.49	0.34 ± 0.24*	3.00 ± 1.33*
*Srebp2*	1.18 ± 0.56	0.41 ± 0.11*	1.58 ± 0.47*
*Insig1*	2.66 ± 2.19*	0.96 ± 0.42	1.36 ± 0.53
*Insig2a*	8.34 ± 5.54*	1.43 ± 0.32*	2.78 ± 1.12*
*Insig2b*	0.24 ± 0.11*	0.74 ± 0.20	0.37 ± 0.20*
*Cyp7a1*	1.29 ± 1.74	0.85 ± 0.44	0.81 ± 0.64
*Cyp8b1*	0.34 ± 0.41*	0.69 ± 0.29	0.43 ± 0.22*
*Cyp2b10*	291.4 ± 194.5*	1.04 ± 0.31	135.4 ± 40.5*
*Cyp3a11*	11.17 ± 6.13*	/	10.8 ± 4.98*

The expression of key genes involved in cholesterol homeostasis and drug metabolism as measured by RT-PCR. Data represents fold change ± standard deviation of TCPOBOP treated versus vehicle treated on standard diet, cholesterol diet, and vehicle treated group on cholesterol diet versus standard diet. Six animals per group were analyzed and statistical significance was determined using Student t-test. Unigene symbols are used as abbreviations. *p < 0.05.

#### Diet-induced hyperlipidemia

One week of 1% cholesterol diet, as expected, increased total cholesterol, HDL-, LDL-cholesterol, total triglycerides and glucose levels (Table 1). The diet also increased total liver cholesterol, resulting in repression of cholesterol biosynthesis observed on the metabolite (Figure 1A and 1B, dark grey boxes), and transcriptome levels (microarrays – Table 2; RT-PCR – Table 3). Also *Srebp2 *and LDL receptor were down-regulated as a result of high liver cholesterol. No changes were detected in primary, CYP7A1-mediated bile acid synthesis pathway on metabolite (Figure 1C, dark grey boxes) or transcriptome levels. However, bile acid transporters on basolateral and canalicular membranes were up-regulated (*Abcb11*, *Slco2b1*).

#### TCPOBOP in diet-induced hyperlipidemia

TCPOBOP treatment of hyperlipidemic animals has significantly lowered serum total cholesterol levels, by lowering the LDL (Table 1). Liver transcriptome data show up-regulation of LDL-receptor and enzyme for cholesterol ester break-down (*Lip1*), and lower ester formation by *Acat2 *(Table 2). However, levels of HDL-cholesterol remain unchanged (Table 1), even if transcriptome data revealed down-regulation of genes connected to HDL particles (*Scarb1*, *Abca1*, *Apoa5*, *Apoe*, and *Saa*), except for *Apoa4*. TCPOBOP lowered the total liver cholesterol of cholesterol-fed mice back to pre-diet levels (Figure 1A, dark grey boxes). This result indicates that TCPOBOP can induce removal of excess dietary cholesterol from the liver and serum in mice. We again see increased primary CYP7A1 pathway of bile acid synthesis on the metabolite level, as indicated by C4 and cholesterol ratio (Figure 1C, dark grey boxes), and no change in expression of *Cyp7a1 *(Table 3). We also see an increase in alternative bile acid synthesis pathways (*Cyp27a1*, *Cyp39a1*), and down-regulation of *Cyp8b1*, all leading to possible formation of more hydrophilic muricholic bile acids. Down-regulation of cholesterol transporter from liver to bile (*Abcg5/Abcg8*) is also observed, again potentially affecting the composition of the bile (Table 2). Controversially, under these high-cholesterol conditions, TCPOBOP has up-regulated the liver cholesterol synthesis as measured by lathosterol and cholesterol ratio (Figure 1B, dark grey boxes) and transcriptome levels (microarrays – Table 2; RT-PCR – Table 3).

**Figure 2:**
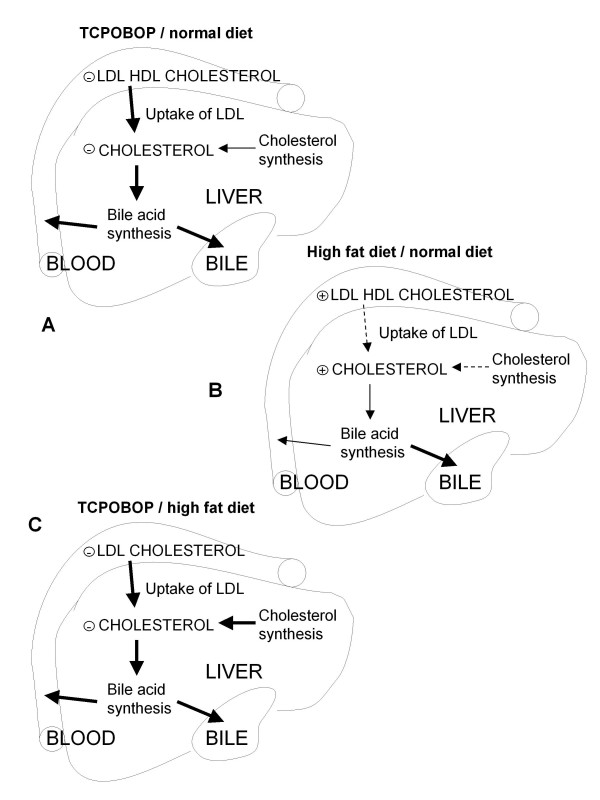
**Effects of TCPOBOP and cholesterol diet on liver cholesterol homeostasis**. Effects of TCPOBOP and cholesterol diet on liver cholesterol metabolism. A. Effects of TCPOBOP in conditions of normal diet. B. Effects of 1 week 1% cholesterol diet. C. Effects of TCPOBOP in conditions of high-cholesterol diet. Bold solid arrow – increase, solid arrow – no change, dashed arrow – decrease.

*In summary *(Figure 2), CAR activation by TCPOBOP leads to lower total serum cholesterol due to activation of the LDL uptake from the circulation and repression of HDL export. The paradoxical drop in liver cholesterol can be explained by activation of the bile acid synthesis and activated pathways for bile efflux from the liver, enabling removal of excess liver cholesterol. Even if the cholesterol diet leads to increased serum and liver cholesterol and the expected decrease in cholesterol synthesis capacity, TCPOBOP is able to reverse these processes. A list of all differentially expressed genes is available in Additional file 1 and Additional file 2 is a more detailed figure depicting TCPOBOP effects on mouse liver under normal and 1% cholesterol-containing diet.

### Triglyceride and glucose metabolism

#### The role of TCPOBOP

The xenobiotic increased total serum triglycerides, but had no effect on glucose levels (Table 1). Transcriptome data are in agreement with elevated triglycerides (Table 4). They indicate up-regulation of fatty acid synthesis (*Fasn*) and ketogenesis (*Hmgcl*), down-regulation of degradation of fatty acids (*Cpt1a*), and up-regulation of the gene responsible for the formation of acetyl-CoA (*Pdhb*), a substrate for cholesterol and fatty acid synthesis. Synthesis of hepatic glycogen is down-regulated (the most down-regulated gene was *Ppp1r3c*, which is imperative for expression of glycogenic enzymes). This also holds for gluconeogenesis (*Pck1*), while glucose transport (*Slc2a1*, increased; and *Slc2a4*, decreased) is modulated. Several genes involved in the regulation of glucose and fatty acid homeostasis were down-regulated (*Adipor2*, *Foxa2*, *Sirt1*, *Cebpa*, *Igf1*, *Ppara*, *Pparg*), but *Chrebp *(*Mlxipl*) was up-regulated.

**Table 4:** Differentially expressed genes involved in glucose and triglyceride metabolism in mouse liver after TCPOBOP treatment

		**TCPOBOP + normal chow diet**		**Vehicle + cholesterol diet**		**TCPOBOP + cholesterol diet**	
**Gene Symbol**	**Gene Description**	**Log_2 _ratio**	**p-value**	**Log_2 _ratio**	**p-value**	**Log_2 _ratio**	**p-value**

**Carbohydrate metabolism**							
Pck1	Phosphoenolpyruvate carboxykinase 1, cytosolic	-0.47	0.042	nc	nc	-0.39	0.039
Pdhb	Pyruvate dehydrogenase (lipoamide) beta	0.61	0.000	0.33	0.005	0.43	0.014
G6Pase	Glucose-6-phosphatase catalytic	nc	nc	1.00	0.005	-1.26	0.000
Gapdh	Glyceraldehyde-3-phosphate dehydrogenase	nc	nc	0.49	0.016	nc	nc
**Fatty acid metabolism**							
Cpt1a	Carnitine palmitoyltransferase 1a, liver	-0.81	0.000	nc	nc	-0.38	0.004
Hmgcl	3-hydroxy-3-methylglutaryl-Coenzyme A lyase	0.31	0.008	-0.53	0.001	0.64	0.003
Fasn	Fatty acid synthase	0.58	0.007	nc	nc	nc	nc
Scd1	Stearoyl-Coenzyme A desaturase 1	nc	nc	1.40	0.004	-1.32	0.018
Cyp4f14	Cytochrome P450, 4f14	nc	nc	0.98	0.000	-0.72	0.000
**Transcription regulators**							
Cebpa	CCAAT/enhancer binding protein (C/EBP), alpha	-1.17	0.000	nc	nc	-0.99	0.000
Sirt1	Sirtuin 1, silent mating type information regulation 2, homolog 1	-0.40	0.010	nc	nc	nc	nc
Foxa2	Forkhead box A2	-0.22	0.049	nc	nc	nc	nc
Mlxipl	MLX interacting protein-like, old Wbscr14 or Chrebp	0.94	0.000	1.05	0.000	0.32	0.047
Crem	CAMP responsive element modulator	nc	nc	0.35	0.002	-0.35	0.016
Ppara	Peroxisome proliferator activated receptor alpha	-0.84	0.000	0.53	0.035	-0.93	0.000
Pparg	Peroxisome proliferator activated receptor gamma	-0.83	0.005	nc	nc	nc	nc
**Transporters**							
Slc2a4	Solute carrier family 2 (facilitated glucose transporter), member 4	-0.43	0.000	nc	nc	nc	nc
Slc2a1	Solute carrier family 2 (facilitated glucose transporter), member 1	0.37	0.001	nc	nc	0.32	0.011
**Cell signaling**							
Ppp1r3c	Protein phosphatase 1, regulatory (inhibitor) subunit 3C	-3.47	0.000	-0.39	0.015	-2.64	0.000
Igf1	Insulin-like growth factor 1	-1.08	0.000	0.69	0.000	-1.37	0.000
Adipor2	Adiponectin receptor 2	-0.85	0.000	nc	nc	-0.80	0.001

Differentially expressed genes involved in glucose and triglyceride metabolism in mouse liver after TCPOBOP treatment or cholesterol diet as detected by the Steroltalk microarray. Data represent log_2 _ratios of TCPOBOP treated versus vehicle treated on the same diet (standard or cholesterol) and vehicle treated group on cholesterol diet versus standard diet. Six animals per group were used and p-value was calculated using t-test as implemented in BRB-Array Tools. GeneBank accession numbers and are available in Additional file 1. nc – no change.

#### Diet-induced hyperlipidemia

Serum metabolite measurements show that the cholesterol diet increased triglycerides and glucose (Table 1), again in agreement with the liver transcriptome data (Table 4). Strong up-regulation of stearoyl-CoA desaturase 1 (*Scd1*) and *Ppara *was measured, which indicates activation of the PPAR*α *signaling pathway. Up-regulated was also the leukotriene B4 omega-hydroxylase (*Cyp4f14*), indicating an increase in fatty acid metabolism. Gluconeogenesis (*G6Pase*, *Gapdh*) was up-regulated while ketogenesis (*Hmgcl*) and glycogenesis (*Ppp1r3c*) were down-regulated.

#### TCPOBOP in diet-induced hyperlipidemia

In mice fed a 1% cholesterol-containing diet, TCPOBOP had no effect on total serum triglycerides but lowered the serum glucose (Table 1). Liver transcriptome analysis (Table 4) indicated up-regulation of the gene involved in formation of acetyl-CoA (*Pdhb*) and ketogenesis (*Hmgcl*), no change in fatty acid synthesis (*Fasn*), and down-regulation of fatty acid metabolism (*Scd1, Cpt1a, Ppara, Cyp4f14*). Glycogen synthesis (*Ppp1r3c*) and gluconeogenesis (*Pck1, G6pase*) were down-regulated and the glucose transporter (*Slc2a1*) up-regulated. Again, genes involved in regulation of liver glucose metabolism (*Igf1, Crem, Cebpa, and Adipor2*) were down-regulated with exception of *Chrebp*, which was up-regulated.

**Figure 3:**
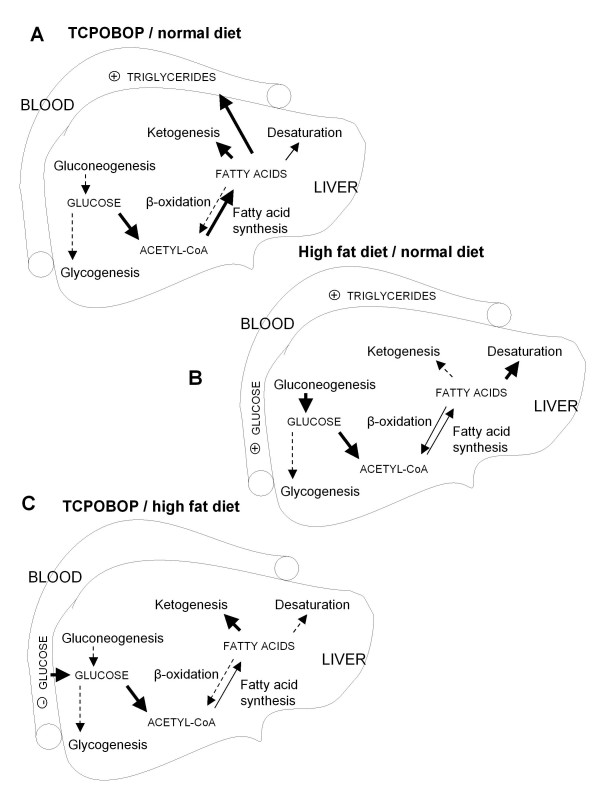
**Effects of TCPOBOP and cholesterol diet on liver glucose and triglyceride metabolism**. Proposed effects of TCPOBOP and cholesterol diet on liver glucose and triglyceride metabolism. A. Effects of TCPOBOP in conditions of normal diet. B. Effects of 1 week 1% cholesterol diet. C. Effects of TCPOBOP in conditions of high-cholesterol diet. Bold solid arrow – increase, solid arrow – no change, dashed arrow – decrease.

Figure 3 summarizes the observed changes. CAR activation by TCPOBOP results in an increase of serum triglycerides due to increased hepatic fatty acid synthesis and lower fatty acid degradation. Even if the decrease in serum glucose was not statistically significant, reduced hepatic *de novo *glycogen and glucose synthesis is observed. A cholesterol diet leads to increased triglycerides and glucose, due to increased liver fatty acid metabolism, repressed glycogen synthesis and activated gluconeogenesis. TCPOBOP is again able to reverse the effect of the cholesterol diet. On the level of triglycerides there is no change in the serum and the hepatic fatty acid synthesis pathways are activated and fatty acid degradation repressed. Serum glucose has dropped due to the repressed gluconeogenesis and activated glucose import.

### Data mining approaches

*Gene annotations *using GeneCodis and KEGG pathways were used to evaluate the expert knowledge based conclusions described above. For the **TCPOBOP **treatment, an effect of CAR activation on *Adipocytokine*, *PPAR *and *Insulin signaling pathways *has been exposed (Table 5). Pathways *Metabolism of xenobiotics by cytochrome P450*, *ABC transporters, Arachidonic acid*, and *Linoleic acid metabolism *were also significant due to TCPOBOP induction of drug metabolism. **Hyperlipidemia **strongly exposed only *Biosynthesis of steroids*. Minor effects were repression of certain enzymes in pathway of *Metabolism of xenobiotics by cytochromes P450*, and importantly, activation of *PPAR signaling pathway*, which is an adaptation to the diet. **TCPOBOP in diet-induced hyperlipidemia **exposed pathway *Biosynthesis of steroids *as a result of up-regulation of cholesterol biosynthesis genes. Again we see the effect on *PPAR *and *Adipocytokine signaling*. Inhibition of *PPAR signaling *represents a response to liver the adaptation on dietary lipids. Pathways of drug metabolism were also exposed.

**Table 5:** Gene annotation of differentially expressed genes

**KEGG pathway**	**No of genes**	**Genes**
**TCPOBOP in normal mouse liver**		
Adipocytokine signaling pathway	10	*Pck1, Adipor1, Ppara, Mapk9, Cpt1a, Slc2a1, Nfkbia, Stat3, Adipor2, Slc2a4*
Metabolism of xenobiotics by cytochrome P450	8	*Cyp1a2, Cyp2b13, Cyp2b10, Cyp2c40, Cyp2e1, Cyp2b9, Cyp3a25, Cyp3a13*
PPAR signaling pathway	7	*Pck1, Cyp8b1, Ppara, Apoa5, Cpt1a, Cyp27a1, Pparg*
ABC transporters – General	6	*Abcc2, Abcb11, Abca1, Abcc1, Abcb4, Abcc3*
Complement and coagulation cascade	6	*Hc, C2, C4bp, Serping1, C9, Fgb*
Insulin signaling pathway	5	Pck1, Fasn, Mapk9, Ppp1r3c, Slc2a4
Arachidonic acid metabolism	5	*Cyp2b13, Cyp2b10, Cyp2c40, Cyp2e1, Cyp2b9*
Linoleic acid metabolism	5	*Cyp1a2, Cyp2c40, Cyp2e1, Cyp3a25, Cyp3a13*
Biosynthesis of steroids	4	*Lss, Sqle, Hmgcr, Sc5d*
**Cholesterol diet**		
Biosynthesis of steroids	13	*Dhcr7, Lss, Mvk, Nsdhl, Idi1, Cyp51a1, Sqle, Hmgcr, Mvd, Fdps, Fdft1, Dhcr24, Sc4mol*
Metabolism of xenobiotics by cytochrome P450	4	*Cyp2f2, Cyp2c40, Cyp3a25, Cyp3a13*
PPAR signaling pathway	4	*Ppara, Scd1, Scp2, Apoc3*
Terpenoid metabolism	4	*Idi1, Sqle, Fdps, Fdft1*
**TCPOBOP in hyperlipidemic mouse liver**		
Biosynthesis of steroids	10	*Lss, Mvk, Nsdhl, Cyp51a1, Hmgcr, Mvd, Fdps, Fdft1, Dhcr24, Sc4mol*
PPAR signaling pathway	7	*Pck1, Cyp8b1, Ppara, Apoa5, Scd1, Cpt1a, Cyp27a1*
Metabolism of xenobiotics by cytochrome P450	7	*Cyp1a2, Cyp2b13, Cyp2b10, Cyp2c40, Cyp2b9, Cyp3a25, Cyp3a13*
Adipocytokine signaling pathway	6	*Pck1, Ppara, Cpt1a, Slc2a1, Adipor2, Mapk8*
Arachidonic acid metabolism	6	*Cyp2b13, Cyp2b10, Cyp2c40, Cyp2b9, Cyp4f14*
ABC transporters – General	5	*Abcg5, Abcc2, Abca1, Abcc3, Abcg8*
Linoleic acid metabolism	5	*Pla2g6, Cyp1a2, Cyp2c40, Cyp3a25, Cyp3a13*
Complement and coagulation cascade	5	*C2, C4bp, Serping1, C9, Fgb*
Butanoate metabolism	4	*Hmgcl, Hmgcs1, Acat2, Pdhb*

Gene annotation of differentially expressed genes using GeneCodis software and KEGG pathways, showing only pathways with at least four genes. Down-regulated genes are underlined.

### Expression of cholesterogenic genes depends on CAR but not SREBP2

**Figure 4:**
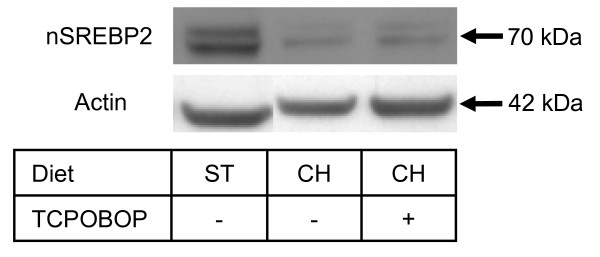
**Representative immunoblot analysis of nuclear SREBP2 in mouse liver**. Representative immunoblot analysis of nuclear SREBP2 in the liver of mice on standard or cholesterol diet and treated with TCPOBOP. No activation of SREBP2 was observed after TCPOBOP application. Diet: ST – standard diet, CH – cholesterol diet.

TCPOBOP has up-regulated SREBP2 target genes in mice fed a 1% cholesterol-containing diet for 1 week. This was unexpected since, under excess cholesterol, the cholesterol feedback loop should prevent proteolytic cleavage and activation of SREBP2. To check whether TCPOBOP would influence proteolytic activation of SREBP2, immunoblot analysis was performed. Figure 4 shows that TCPOBOP and subsequent CAR activation did not increase the SREBP2 active form in the nucleus. We therefore propose that the observed TCPOBOP up-regulation of cholesterogenic genes is SREBP2 independent.

To evaluate whether the up-regulation of SREBP2 responsive genes by TCPOBOP might be a direct effect of CAR, RT-PCR analyses in livers of wild type and CAR knock-out mice fed with standard chow diet were performed (Table 6). Three cholesterogenic genes (*Hmgcr*, *Cyp51a1 *and *Sqle*) and two genes from the SREBP signaling pathway (*Insig1, Srebp2*) were selected while *Cyp3a11 *and *Cyp2b10 *served as positive controls for the CAR regulated genes. In wild-type mice TCPOBOP increased the expression of *Hmgcr*, *Insig1 *and *Sqle *while expression of *Cyp51a1 *and *Srebp2 *remained unchanged. TCPOBOP in CAR deficient mice lowered expression of all measured genes. Comparison of CAR -/- mice to wild-type showed a higher basal expression of SREBP2-dependent genes. These results give two possible hypotheses: CAR without the ligand might serve as a direct or indirect regulator of these genes. Since expression analysis indicated the possibility of direct regulation of SREBP-2 dependent genes by CAR, analysis of *Hmgcr*, *Sqle *and *Insig1 *promoters was performed to search for potential CAR binding sites. Using specialized software for nuclear receptor binding site search NUBIScan [21] we found no CAR (DR4, DR3, ER6) binding sites in *Hmgcr*, *Sqle *promoters. However, a DR4 site in *Insig1 *promoter was found, which was already confirmed experimentally [22].

**Table 6:** Expression of genes in knock-out mice treated with TCPOBOP as detected by RT-PCR

**Treatment**	**Gene name**	**Genotype**	
		**WT**	**CAR -/-**

**TCPOBOP**	***Hmgcr***	2.22 ± 1.38	0.35 ± 0.18*
	***Sqle***	2.18 ± 1.1*	0.69 ± 0.25*
	***Cyp51a1***	1.43 ± 0.89	0.43 ± 0.16*
	***Insig1***	1.86 ± 0.77	0.55 ± 0.24*
	***Srebp2***	1.31 ± 0.73	0.49 ± 0.20*
	***Cyp3a11***	5.82 ± 3.35*	0.44 ± 0.36
	***Cyp2b10***	5750 ± 2805*	3.59 ± 5.61

**Untreated CAR -/- *vs *WT**	***Hmgcr***	-	4.1 ± 1.26*
	***Sqle***	-	2.90 ± 0.55*
	***Cyp51a1***	-	2.81 ± 0.74*
	***Insig1***	-	3.38 ± 1.02*
	***Srebp2***	-	3.95 ± 1.14*
	***Cyp3a11***	-	3.03 ± 1.80
	***Cyp2b10***	-	0.76 ± 0.54

The effect of TCPOBOP and CAR on the expression of cholesterol synthesis and Srebp2 signaling genes as measured by RT-PCR. Cyp3a11 and Cyp2b10 serve as positive controls for CAR activated genes. Data represents fold changes ± standard deviation of treated versus vehicle treated or untreated CAR -/- versus wild type (WT). All animals were fed standard chow diet. Six animals per group were analyzed and statistical significance was determined using Student t-test. Unigene symbols were used as abbreviations. *p < 0.05.

## Discussion

Hyperlipidemias provoke cardiovascular diseases that represent a major cause of deaths in the developed world. Studies in recent years show that metabolic disturbances in the liver contribute to metabolic diseases (*i.e*. diabetes, nonalcoholic fatty liver disease (NAFLD)) and cardiovascular diseases. Nuclear receptors, such as PPARs, LXR and FXR, are important regulators of the metabolic network and are the targets of new therapeutic agents for treatment of metabolic disorders [23]. Increasing amounts of data also indicate that constitutive androstane receptor CAR plays a role in the development of metabolic disorders [24].

In the present study, we investigated CAR activation by TCPOBOP in diet-induced hyperlipidemia in the liver by using a dedicated microarray approach, liver and serum sterol measurements. CAR activation by TCPOBOP resulted in systemic dyslipidemia that includes lower serum LDL and HDL cholesterol and increased triglycerides (Additional file 2). Lower LDL-cholesterol and total plasma cholesterol are considered beneficial effects of CAR activators that result from increased liver uptake of lipoproteins by up-regulated LDLR. In addition, liver pathways for the bile acid synthesis and their removal are up-regulated, contributing to decreased serum and liver cholesterol. We observed up-regulation of CYP7A1 pathway, but only on metabolite level and not on RNA level. This discrepancy is probably due to large inter-individual variations in CYP7A1 expression which affects statistical significance. It seems that TCPOBOP activates both CYP7A1 and CYP27A1 pathways; however, without a detailed analysis of bile acid content it is not possible to conclude to what extent each pathway is affected. In diet-induced hyperlipidemia, which represents a lipid metabolic disturbance *per se*, CAR activation by TCPOBOP lowers total cholesterol and LDL, but not HDL cholesterol, whereas triglycerides remain elevated. The cholesterol diet by itself did not change the expression of the bile acid pathways, while application of TCPOBOP led to similar changes as in the control liver (Additional file 2). As a consequence, in normal and hyperlipidemic livers this may result in altered composition of bile. A parameter of dyslipidemia is also the TCPOBOP-mediated repression of cholesterol influx from HDL to the liver in normal and hyperlipidemic mice, where HDL-cholesterol levels remain elevated. The influence of TCPOBOP on formation of HDL was shown by another study, where CAR-dependent decrease in HDL was linked to repression of ApoA-I synthesis [19]. In our study, several acute phase proteins, such as serum amyloids (Saa) and apolipoprotein A-V, were down-regulated, while apolipoprotein A-IV was up-regulated. Low-grade inflammation is associated with an increased risk of coronary events. The marker of this is CRP (C-reactive protein) the expression of which was increased in TCPOBOP treated normal and hyperlipidemic mice. However, whether the changes induced by TCPOBOP are pro-atherogenic remains unknown.

The metabolic syndrome increases the risk for development of cardiovascular diseases, and *vice versa*. Nonalcoholic steatohepatitis (NASH) is a nonalcoholic fatty liver disease (NAFLD). It develops from liver steatosis in the presence of oxidative stress, insulin resistance, hepatotoxins and other factors [25]. A study of diet induced NASH in mice showed that CAR activation by TCPOBOP increased lipid peroxidation and oxidative stress, and thereby contributes to the development of NASH [17]. Another aspect of CAR activation and development of NAFLD is the effect of CAR on adiponectin, an adipocyte hormone, and on the PPARα signaling pathway as indicated by pathway analysis of our transcriptome data in normal and hyperlipidemic liver. Adiponectin can prevent the development of diabetes and metabolic syndrome, as it stimulates glucose utilization and fatty acid combustion in the liver [26]. Our transcriptome analysis indeed reveals that several genes of the adiponectin signaling are modulated. For instance, the adiponectin receptor II was down-regulated, a phenomenon also observed in patients with NASH [27]. Adiponectin stimulation of fatty acid degradation is mediated by PPARα and CAR inhibition of this pathway might further contribute to promotion of a metabolic syndrome. More importantly, up-regulation of the PPARα signaling pathway is also an adaptation of the liver to hyperlipidemia, and TCPOBOP disturbed this adaptation. CAR also increased expression of *Chrebp*, which stimulates fatty acid synthesis [28]. Our data thus indicate that CAR activation in some aspects mimics the effect of insulin on the liver. CAR, similarly to insulin, inhibits liver fatty acid oxidation, gluconeogenesis and stimulates fatty acid synthesis and glycolysis. However, unlike insulin, CAR activation represses glycogen synthesis. It therefore seems that CAR stimulates glucose metabolism to acetyl-CoA, which can have a positive effect on elevated plasma glucose levels, as seen in hyperlipidemic mice. However, acetyl-CoA is used later for fatty acid and cholesterol synthesis. Elevated cholesterol biosynthesis does not result in higher liver or plasma levels, because CAR also up-regulates cholesterol removal pathways. However, increased fatty acid synthesis and repressed oxidation represent an important contribution of CAR to the development of NAFLD.

Interestingly, TCPOBOP-induced CAR also up-regulated cholesterol synthesis in hyperlipidemic liver and affected the SREBP signaling pathway [29]. Our transcriptome data indicate that TCPOBOP activates the group of SREBP2 regulated genes; however, immunoblot analysis revealed no increase in the nuclear SREBP2. This might result from a CAR-dependent up-regulation of *Insig1*, which inhibits the SREBP2 cleavage [22]. RT-PCR analysis of CAR knock-out animals treated with TCPOBOP indicate that up-regulation of cholesterogenic genes and the SREBP signaling pathway is CAR-dependent. Since no CAR binding sites were found in the two most responsive genes, HMG-CoA reductase (*Hmgcr*) and squalene epoxidase (*Sqle*), mechanisms other than direct transcriptional activation by CAR have to be considered. CAR can activate another common transcription factor, different from SREPB2, which can bind to proximal promoters of cholesterogenic genes. Alternatively, a non-activated CAR could act as a repressor of cholesterogenic genes. Again, in the absence of true CAR binding sites in promoters of these genes it is difficult to draw conclusions. However, it was reported most recently that SF3a3 functions as a co-repressor of CAR transcriptional activity independently of the presence of TCPOBOP [30].

## Conclusion

Activation of CAR by TCPOBOP alters hepatic metabolism in the livers of mice fed normal and cholesterol-containing diets. TCPOBOP increases removal of excess dietary cholesterol in serum and liver, and lowers serum glucose and liver gluconeogenesis under conditions of hyperlipidemia. On the other hand, CAR activation increases serum triglycerides and liver fatty acid synthesis, represses adaptation to hyperlipidemia, and lowers responsiveness of the liver to adiponectin. These latter effects favor the development of non-alcoholic fatty liver disease. Finally, CAR affects downstream SREBP2 signaling (without changing nuclear SREBP2 protein levels) and is thereby involved in the regulation of cholesterol synthesis.

## Methods

### Mouse experiment and preparation of liver RNA samples

All animal experiments followed the Amsterdam Protocol on Animal Protection and Welfare and were approved by the local ethics committee. Four groups (6 animals per group) of female mice C57BL/6 (Harlan) age between 18 and 19 weeks were housed in normal light-cycle room, maintained on standard rodent chow (diet 3430, Provimi Kliba SA, Kaiseraugst, CH), and allowed water and food *ad libitum*. One week prior to treatment, two groups of mice were switched to a 1% (w/w) cholesterol diet (diet Western 24769, Provimi Kliba SA, Kaiseraugst, CH). One group of mice from each diet were injected i.p. with either vehicle (corn oil, Sigma, St Louis, MI, USA) or with 3 mg/kg TCPOBOP (Bayer Ag, Wuppertal, Germany) in corn oil. After 24 hours, mice were sacrificed using CO_2 _suffocation. Blood was transferred into tubes (BD Biosciences-Pharmingen) and after centrifugation, serum was stored at -80°C. Left lateral lobes of livers were snap frozen in liquid nitrogen and stored at -80°C. Total RNA was isolated from liver using TRI-reagent (Sigma, St Louis, MI, USA) according to the manufacturer's protocol and purified by ethanol precipitation.

Experiments with CAR-/- mice were performed as described [22]. Briefly, male C57BL/6J wild type or CAR -/- mice, age 9–12 weeks, were maintained on standard laboratory chow and had free access to food and water. They were maintained in 12-hour light/dark cycle. Six mice per group were injected i.p. with vehicle (5%DMSO in corn oil) or with 10 mg/kg TCPOBOP (Bayer Ag, Wuppertal, Germany). After 12 hours mice were sacrificed and total liver RNA was isolated using TRIzol reagent (Invitrogen, Carlsbad, CA, USA).

### Microarray analyses

The Steroltalk cDNA microarrays were prepared as previously described [31]. Total RNA from each animal was labeled separately, and a reference sample was prepared by pooling all RNA samples in the study in equal amounts. To each RNA sample a spike in RNA was added and transcribed to Cy3 or Cy5 labeled cDNA using amino–allyl labeling. Details of the microarray experiment are described in Additional file 3. Classification of differentially expressed genes was done in BRB-Array Tools Version 3.7.0 beta_2 release developed by Dr. Richard Simon and Amy Peng Lam using Class comparison between groups and significance threshold α = 0.05 and FDR<0.16. Genes were further annotated using GeneCodis and KEGG pathways [32]. All data have been deposited in GEO database, reference number GSE13688.

### Real-time polymerase chain reaction analyses

In studies of diet effect on TCPOBOP activation, analyses were performed using Platinum^® ^SYBR^® ^Green qPCR SuperMix-UDG (Invitrogen, Carlsbad, CA, USA) on ABI PRISM 7900 HT (PE Applied Biosystems, Foster City, CA, USA) according to manufacturer's protocols. In samples from knock-out livers, analyses were performed using LightCycler^® ^480 SYBR Green I Master on LightCycler^® ^480 (Roche Diagnostics GmbH, Mannheim, Germany), except *Cyp2b10*, which was done as described above. 18S rRNA served as internal control in both studies. Relative transcript levels were calculated by the comparative Ct (cycle threshold) method and -ΔΔCt values were used for statistical analyses [33]. Levene's test for equality of variance was performed, and Student t-test and a probability of type I error α = 0.05 was used to determine statistical significance in SPSS 14.0 (SPSS Inc., Chicago, Illinois, USA). Primer sequences and analysis details are provided in Additional file 3.

### Liver sterol analyses

Two frozen liver samples from individual animals were combined yielding three pools per treatment and sterol metabolites were isolated and analyzed as described elsewhere [34]. Levels of free lathosterol, C4 and total cholesterol were measured using GC-MS. Lathosterol is an intermediate of cholesterol biosynthesis and has been shown to correlate well with the activity of HMG-CoA reductase [35]. Free lathosterol/total cholesterol ratio was therefore used as a marker of metabolic rate of liver cholesterol biosynthesis. C4 is an intermediate of classical bile acid pathway and can be produced only by enzymatic activity (Björkhem I, personal communications). The quantity of measured sterols (ng/mg liver) was calculated using standard curve, and these values were used for statistical analyses. Details are available in Additional file 3. Levene's test for equality of variance was performed, and Student t-test and a probability of type I error α = 0.05 were used to determine statistical significance in SPSS 14.0 (SPSS Inc., Chicago, Illinois, USA). In the case of C4/cholesterol, ratio variance was not equal and so we applied Mann-Whitney U test for statistical significance.

### Measurements of serum parameters

Total serum cholesterol and triglyceride analyses were performed on RX Daytona analyser by the enzymatic colometric method using Cholesterol CHOD/PAP (Dimension^®^) and Triglycerides GPO/PAP kit (Dimension^®^) (Randox Laboratories Ltd., Crumlin, UK), respectively. HDL and LDL cholesterol levels were analyzed by Cobas Mira analyser and direct enzymatic method (Randox Laboratories Ltd, Crumlin, UK). Glucose was measured by UV test using hexokinase and glucose 6-phosphate dehydrogenase (Randox Laboratories Ltd., Crumlin, UK) using Cobas Mira analyser. All analyses were done at Veterinary Faculty, University of Ljubljana.

### Immunoblot analyses of nuclear Srebp2

Liver samples from two animals were pooled, yielding 3 biological replicas per treatment and nuclear proteins were extracted (details see in Additional file 3). Proteins were analyzed using SDS-PAGE gels NuPAGE^® ^Novex 4–12% Bis-Tris Gels and run using XCellTM Surelock Mini-Cell (Invitrogen, Carlsbad, CA, USA) according to manufacturer's protocol. Proteins were transferred to PVDF membrane HybondTM-P (Amersham Biosciences, GE Healthcare UK limited, Little Chalfont, UK) using XCell IITM Blot Module and NuPAGE^® ^Transfer Buffer (Invitrogen, Carlsbad, CA, USA) according to manufacturer's protocol. Mouse anti-Srebp2 (BD Biosciences San Jose, CA, USA) and anti-actin (Sigma, St. Louis, MI, USA) antibodies were used.

### Bioinformatical analysis of promoters

Genomic sequences from exon 2 to 10 kb distal promoter were selected and analyzed by NUBIScan software, searching for DR4, DR3 and ER6 sites with p-value cut-of at 0.05 [21].

## Authors' contributions

TR performed animal experiment on wild type mice, RNA extractions, transcriptome analysis, all RT-PCR analysis, protein isolations and western blot analysis, statistical analysis, and wrote the manuscript. VT performed knock-out animal experiments and RNA isolations under supervision of UAM. UAM also contributed to the overall idea of the study. ALS performed sterol extractions and analysis under supervision of IB. DR contributed to the overall idea and design of the study, and was supervisor of TR. All authors have read and approved the final manuscript.

## Supplementary Material

Additional file 1 **Differentially expressed genes in mouse liver after TCPOBOP treatment**. Differentially expressed genes in mouse liver after TCPOBOP treatment or cholesterol diet as detected by the Steroltalk microarray. Data represent log_2 _ratios of TCPOBOP treated versus vehicle treated on the same diet (standard or cholesterol) and vehicle treated group on cholesterol diet versus standard diet. Six animals per group were used and p-value was calculated using t-test as implemented in BRB-Array Tools. nc – no change. Click here for file

Additional file 2 **Systemic effects of TCPOBOP on liver metabolism**. Systemic effects of TCPOBOP on liver metabolism. A. Effects of TCPOBOP in conditions of normal diet. B. Effects of 1 week 1% cholesterol diet. C. Effects of TCPOBOP in conditions of high-cholesterol diet. Bold genes, metabolites or processes are up-regulated. Click here for file

Additional file 3 Additional data about materials and methods. Click here for file
