# Supplementary material for: Effect of CAR activation on selected metabolic pathways in normal and hyperlipidemic mouse livers
Source: BMC Genomics. 2009 Aug 19;10:384. doi: 10.1186/1471-2164-10-384 (PMC2739862; doi:10.1186/1471-2164-10-384)
Supplement: Additional file 1 — Differentially expressed genes in mouse liver after TCPOBOP treatment. Differentially expressed genes in mouse liver after TCPOBOP treatment or cholesterol diet as detected by the Steroltalk microarray. Data represent log2 ratios of TCPOBOP treated versus vehicle treated on the same diet (standard or cholesterol) and vehicle treated group on cholesterol diet versus standard diet. Six animals per group were used and p-value was calculated using t-test as implemented in BRB-Array Tools. nc – no change. [file 1471-2164-10-384-S1.pdf]

Differentially expressed genes in mouse liver after TCPOBOP treatment

Differentially expressed genes in mouse liver after TCPOBOP treatment or cholesterol diet as detected by the Steroltalk microarray. Data represent log_2 ratios of TCPOBOP treated versus vehicle treated on the same diet (standard or cholesterol) and vehicle treated group on cholesterol diet versus standard diet. Six animals per group were used and p-value was calculated using t-test as implemented in BRB-Array Tools. nc – no change.

			TCPOBOP		TCPOBOP + cholesterol diet		Cholesterol diet	
**Gene Symbol**	**GenBank Accession**	**Gene Description**	**Log_2 ratio**	**p-value**	**Log_2 ratio**	**p-value**	**Log_2 ratio**	**p-value**
**Cholesterol biosynthesis**								
Sc4mol	NM_025436	Sterol-C4-methyl oxidase-like	nc	nc	1.85	0.001	-2.50	0.000
Cyp51	BC031813	Cytochrome P450, family 51	nc	nc	1.27	0.008	-1.86	0.001
Hmgcs1	NM_145942	3-hydroxy-3-methylglutaryl-Coenzyme A synthase 1	nc	nc	1.20	0.015	-1.79	0.001
Fdps	NM_134469	Farnesyl diphosphate synthase 1	nc	nc	0.99	0.026	-1.73	0.001
Fdft1	NM_010191	Farnesyl diphosphate farnesyl transferase 1, Squalene synthase	nc	nc	1.08	0.006	-1.69	0.000
Nsdhl	BC019945	NAD(P) dependent steroid dehydrogenase-like	nc	nc	0.99	0.033	-1.35	0.004
Lss	NM_146006	Lanosterol synthase	0.60	0.033	0.81	0.012	-1.31	0.000
Mvd	NM_138656	Mevalonate (diphospho) decarboxylase	nc	nc	0.88	0.017	-1.27	0.002
Mvk	NM_023556	Mevalonate kinase	nc	nc	0.86	0.010	-1.18	0.001
Hmgcr	NM_008255	3-hydroxy-3-methylglutaryl-Coenzyme A reductase	0.99	0.001	1.09	0.008	-1.14	0.001
Sqle	BC042781	Squalene epoxidase	0.49	0.014	nc	nc	-1.11	0.014
Dhcr24	BC019797	24-dehydrocholesterol reductase	nc	nc	0.54	0.018	-0.57	0.019
Dhcr7	BC006854	7-dehydrocholesterol reductase	nc	nc	nc	nc	-0.36	0.049
Idi1	NM_177960	Isopentenyl-diphosphate delta isomerase	nc	nc	nc	nc	0.39	0.003
Sc5d	BC024132	Sterol-C5-desaturase homolog	0.36	0.029	nc	nc	nc	nc
**SREBP signaling pathway**								
Insig1	NM_153526	Insulin induced gene 1	0.82	0.000	nc	nc	nc	nc
Mbtps1	NM_019709	Membrane-bound transcription factor peptidase, site 1	1.72	0.000	2.06	0.003	nc	nc
Insig2	BC023874	Insulin induced gene 2	1.94	0.000	1.24	0.000	0.25	0.038
**Bile acid synthesis**								
Cyp8b1	NM_010012	Cytochrome P450, family 8, subfamily b, polypeptide 1	-2.16	0.000	-0.67	0.036	nc	nc
Cyp7b1	BC038810	Cytochrome P450, family 7, subfamily b, polypeptide 1	0.21	0.014	nc	nc	nc	nc
Cyp27a1	NM_024264	Cytochrome P450, family 27, subfamily a, polypeptide 1	0.73	0.000	0.46	0.028	nc	nc
Cyp39a1	AF237981	Cytochrome P450, family 39, subfamily a, polypeptide 1	1.06	0.000	0.91	0.000	nc	nc

**Lipid transport**								
Apoa5	BC011198	Apolipoprotein A-V	-0.57	0.013	-0.45	0.039	nc	nc
Apoe	BC028816	Apolipoprotein E	-0.38	0.019	-0.46	0.022	nc	nc
Ldlr	BC019207	Low density lipoprotein receptor	0.32	0.017	0.42	0.039	-0.38	0.037
Apoa4	BC010769	Apolipoprotein A-IV	2.23	0.000	1.00	0.001	1.09	0.000
Apoc3	BC021776	Apolipoprotein C-III	nc	nc	nc	nc	0.43	0.006
Apoc1	BC094638	Apolipoprotein C-I	nc	nc	nc	nc	0.28	0.039
**Steroid synthesis**								
Cyp21a1	NM_009995	Cytochrome P450, family 21, subfamily a, polypeptide 1	0.56	0.000	0.57	0.013	nc	nc
Cyp17a1	NM_007809	Cytochrome P450, family 17, subfamily a, polypeptide 1	1.62	0.000	1.03	0.000	nc	nc
**Transporters**								
Abcc1	NM_008576	ATP-binding cassette, sub-family C (CFTR/MRP), member 1	-0.50	0.016	nc	nc	nc	nc
Abcb4	NM_008830	ATP-binding cassette, sub-family B (MDR/TAP), member 4	-0.46	0.001	nc	nc	nc	nc
Slc2a4	BC014282	Solute carrier family 2 (facilitated glucose transporter), member 4	-0.43	0.000	nc	nc	nc	nc
Slco2b1	BC096485	Solute carrier organic anion transporter family, member 2b1	-0.40	0.001	nc	nc	0.25	0.009
Abca1	NM_013454	ATP-binding cassette, sub-family A (ABC1), member 1	-0.39	0.024	-0.38	0.015	0.18	0.040
Slc2a1	BC055340	Solute carrier family 2 (facilitated glucose transporter), member 1	0.37	0.001	0.32	0.011	nc	nc
Abcb1a	NM_011076	ATP-binding cassette, sub-family B (MDR/TAP), member 1A	0.41	0.000	0.31	0.024	nc	nc
Abcb11	NM_021022	ATP-binding cassette, sub-family B (MDR/TAP), member 11	0.46	0.001	nc	nc	0.68	0.000
Abcc2	NM_013806	ATP-binding cassette, sub-family C (CFTR/MRP), member 2	0.92	0.000	0.89	0.000	nc	nc
Slco1a4	NM_030687	Solute carrier organic anion transporter family, member 1a4	1.04	0.000	1.66	0.000	-0.98	0.000
Abcc3	NM_029600	ATP-binding cassette, sub-family C (CFTR/MRP), member 3	1.29	0.000	2.06	0.000	-0.76	0.003
Abcg8	NM_026180	ATP-binding cassette, sub-family G (WHITE), member 8	nc	nc	-1.02	0.006	nc	nc
Abcg5	NM_031884	ATP-binding cassette, sub-family G (WHITE), member 5	nc	nc	-0.87	0.008	nc	nc
Slc10a2	NM_011388	Solute carrier family 10, member 2	nc	nc	0.62	0.039	nc	nc
**Drug metabolism**								
Cyp2e1	NM_021282	Cytochrome P450, family 2, subfamily e, polypeptide 1	-0.67	0.000	nc	nc	nc	nc
Cyp2c40	NM_010004	Cytochrome P450, family 2, subfamily c, polypeptide 40	-0.35	0.015	-0.71	0.003	0.91	0.000
Cyp2a12	NM_133657	Cytochrome P450, family 2, subfamily a, polypeptide 12	0.83	0.000	0.66	0.000	nc	nc
Cyp3a13	NM_007819	Cytochrome P450, family 3, subfamily a, polypeptide 13	1.34	0.000	1.83	0.000	-0.58	0.007
Cyp3a25	BC028855	Cytochrome P450, family 3, subfamily a, polypeptide 25	1.53	0.000	1.80	0.000	-0.64	0.001
Cyp2a4	BC011233	Cytochrome P450, family 2, subfamily a, polypeptide 4	1.86	0.000	1.62	0.000	-0.41	0.010
Cyp1a2	NM_009993	Cytochrome P450, family 1, subfamily a, polypeptide 2	1.90	0.000	1.81	0.000	nc	nc
Cyp2b9	NM_010000	Cytochrome P450, family 2, subfamily b, polypeptide 9	2.30	0.000	2.41	0.000	nc	nc
Cyp2b13	NM_007813	Cytochrome P450, family 2, subfamily b, polypeptide 13	2.89	0.000	2.98	0.000	nc	nc
Cyp2b10	AK028103	Cytochrome P450, family 2, subfamily b, polypeptide 10	3.12	0.000	3.18	0.000	nc	nc
Cyp2g1	NM_013809	Cytochrome P450, family 2, subfamily g, polypeptide 1	nc	nc	0.50	0.008	nc	nc

Cyp2f2	NM_007817	Cytochrome P450, family 2, subfamily f, polypeptide 2	nc	nc	nc	nc	0.48	0.007
**Other**								
Scarb1	NM_016741	Scavenger receptor class B, member 1	-0.87	0.000	-0.88	0.000	0.31	0.003
Orm1	BC012725	Orosomucoid 1	-0.79	0.013	nc	nc	nc	nc
Acat2	NM_009338	Acetyl-Coenzyme A acetyltransferase 2	-0.31	0.010	-0.33	0.019	nc	nc
Gfpt2	BC031928	Glutamine fructose-6-phosphate transaminase 2	-0.29	0.006	nc	nc	nc	nc
Cyp4b1	NM_007823	Cytochrome P450, family 4, subfamily b, polypeptide 1	0.25	0.042	nc	nc	nc	nc
Uap1	BC016406	UDP-N-acetylglucosamine pyrophosphorylase 1	0.42	0.008	0.34	0.039	nc	nc
Cyp26a1	NM_007811	Cytochrome P450, family 26, subfamily a, polypeptide 1	1.98	0.000	1.77	0.000	nc	nc
Scp2	BC018384	Sterol carrier protein 2, liver	nc	nc	nc	nc	0.34	0.005
Icam1	BC008626	Intercellular adhesion molecule	nc	nc	nc	nc	-0.33	0.031
**Nuclear receptor superfamily**								
Ppara	NM_011144	Peroxisome proliferator activated receptor alpha	-0.84	0.000	-0.93	0.000	0.53	0.035
Pparg	NM_011146	Peroxisome proliferator activated receptor gamma	-0.83	0.005	nc	nc	nc	nc
Nr1i3	NM_009803	Nuclear receptor subfamily 1, group I, member 3	-0.79	0.000	nc	nc	-0.54	0.018
Nr2f6	NM_010150	Nuclear receptor subfamily 2, group F, member 6	-0.47	0.007	-0.32	0.031	nc	nc
Nr1i2	NM_010936	Nuclear receptor subfamily 1, group I, member 2	0.29	0.011	0.47	0.015	nc	nc
Nr5a2	NM_030676	Nuclear receptor subfamily 5, group A, member 2	0.34	0.002	nc	nc	0.46	0.001
Nr1d2	NM_011584	Nuclear receptor subfamily 1, group D, member 2	0.70	0.000	1.10	0.003	nc	nc
Nr2f2	NM_009697	Nuclear receptor subfamily 2, group F, member 2	nc	nc	nc	nc	0.38	0.005
**Transcription regulators**								
Cebpa	BC028890	CCAAT/enhancer binding protein (C/EBP), alpha	-1.17	0.000	-0.99	0.000	nc	nc
Sirt1	NM_019812	Sirtuin 1, silent mating type information regulation 2, homolog 1	-0.40	0.010	nc	nc	nc	nc
Sp1	AF062566	Trans-acting transcription factor 1, Specificity protein 1	-0.35	0.002	nc	nc	nc	nc
Pcaf	BC082581	P300/CBP-associated factor	-0.32	0.001	nc	nc	nc	nc
Foxa2	NM_010446	Forkhead box A2	-0.22	0.049	nc	nc	nc	nc
Mlxipl	NM_021455	MLX interacting protein-like, old Wbscr14 or Chrebp	0.94	0.000	0.32	0.047	1.05	0.000
Crem	M60285	CAMP responsive element modulator	nc	nc	-0.35	0.016	0.35	0.002
Foxo1	NM_019739	Forkhead box O1	nc	nc	nc	nc	0.33	0.004
Cebpg	BC011319	CCAAT/enhancer binding protein (C/EBP), gamma	nc	nc	nc	nc	0.27	0.023
Cebpz	NM_009882	CCAAT/Enhancer Binding Protein Zeta	nc	nc	nc	nc	0.31	0.032
**Carbohydrate metabolism**								
Pck1	BC037629	Phosphoenolpyruvate carboxykinase 1, cytosolic	-0.47	0.042	-0.39	0.039	nc	nc
Pdhb	NM_024221	Pyruvate dehydrogenase (lipoamide) beta	0.61	0.000	0.43	0.014	0.33	0.005
G6Pase	NM_008061	Glucose-6-phosphatase catalytic	nc	nc	-1.26	0.000	1.00	0.005
**Fatty acid metabolism**								
Cpt1a	BC038395	Carnitine palmitoyltransferase 1a, liver	-0.81	0.000	-0.38	0.004	nc	nc

Hmgcl	BC025440	3-hydroxy-3-methylglutaryl-Coenzyme A lyase	0.31	0.008	0.64	0.003	-0.53	0.001
Lip1	NM_021460	Lysosomal acid lipase 1	0.49	0.000	0.28	0.043	0.38	0.002
Fasn	BC046513	Fatty acid synthase	0.58	0.007	nc	nc	nc	nc
Scd1	BC007474	Stearoyl-Coenzyme A desaturase 1	nc	nc	-1.32	0.018	1.40	0.004
Cyp4f14	NM_022434	Cytochrome P450, family 4, subfamily f, polypeptide 14	nc	nc	-0.72	0.000	0.98	0.000
Pla2g6	BC003487	Phospholipase A2, group VI	nc	nc	0.92	0.020	nc	nc
**Cell signaling**								
Ppp1r3c	NM_016854	Protein phosphatase 1, regulatory (inhibitor) subunit 3C	-3.47	0.000	-2.64	0.000	-0.39	0.015
Igf1	NM_010512	Insulin-like growth factor 1	-1.08	0.000	-1.37	0.000	0.69	0.000
Adipor2	NM_197985	Adiponectin receptor 2	-0.85	0.000	-0.80	0.001	nc	nc
Serping1	BC002026	Serine (or cysteine) peptidase inhibitor, clade G, member 1	-0.65	0.005	-0.47	0.009	nc	nc
Ogt	BC057319	O-linked N-acetylglucosamine (GlcNAc) transferase (UDP-N-acetylglucosamine:polypeptide-N-acetylglucosaminyl transferase)	-0.51	0.000	nc	nc	nc	nc
Stat3	BC003806	Signal transducer and activator of transcription 3	-0.46	0.013	nc	nc	-0.35	0.028
Camk1d	NM_177343	Calcium/calmodulin-dependent protein kinase ID	-0.32	0.037	-0.32	0.018	nc	nc
Nfkbia	BC046754	Nuclear factor of kappa light chain gene enhancer in B-cells inhibitor, alpha	-0.29	0.041	nc	nc	nc	nc
Mapk9	BC028341	Mitogen activated protein kinase 9	-0.21	0.029	nc	nc	-0.17	0.030
Adipor1	NM_028320	Adiponectin receptor 1	0.19	0.021	nc	nc	nc	nc
Sumo2	BC017522	SMT3 suppressor of mif two 3 homolog 2 (yeast)	0.32	0.005	nc	nc	nc	nc
Mapk8	BC053027	Mitogen activated protein kinase 8	nc	nc	0.40	0.016	nc	nc
Gckr	NM_144909	Glucokinase regulatory protein	nc	nc	nc	nc	0.34	0.023
**Heme metabolism**								
Hpxn	BC019901	Hemopexin	-0.52	0.039	-0.40	0.015	nc	nc
Alas1	NM_020559	Aminolevulinic acid synthase 1	1.81	0.000	2.00	0.000	nc	nc
**Housekeeping genes**								
Ppia	NM_008907	Peptidylprolyl isomerase A	0.39	0.004	0.29	0.043	nc	nc
Actb	NM_007393	Actin, beta, cytoplasmic	0.67	0.010	nc	nc	0.75	0.008
Gapdh	NM_008084	Glyceraldehyde-3-phosphate dehydrogenase	nc	nc	nc	nc	0.49	0.016
**Circadian regulation**								
Cry1	NM_007771	Cryptochrome 1 (photolyase-like)	-0.63	0.000	-0.72	0.000	0.24	0.042
Arntl	BC011080	Aryl hydrocarbon receptor nuclear translocator-like	-0.36	0.003	-0.26	0.038	nc	nc
Clock	AF000998	Circadian locomoter output cycles kaput	0.22	0.009	nc	nc	nc	nc
**Serum proteins**								
Saa2	BC024606	Serum amyloid A 2	-3.60	0.000	-3.28	0.001	nc	nc
Saa1	BC087933	Serum amyloid A 1	-3.52	0.000	-3.22	0.001	nc	nc
Saa3	BC055885	Serum amyloid A 3	-3.39	0.000	-2.87	0.001	nc	nc
C4bp	NM_007576	Complement component 4 binding protein	-1.09	0.000	-0.69	0.001	nc	nc
C9	BC011137	Complement component 9	-0.88	0.000	-1.15	0.000	1.13	0.000
C4b	BC067409	Complement component 4B (Childo blood group)	-0.70	0.000	-0.51	0.001	nc	nc
Fgb	NM_181849	Fibrinogen, B beta polypeptide	-0.67	0.019	-0.28	0.036	-0.58	0.007
C2	BC011086	Complement component 2 (within H-2S)	-0.53	0.008	-0.39	0.017	nc	nc

Hc	M35525	Hemolytic complement	-0.49	0.002	nc	nc	nc	nc
Saa4	BC019212	Serum amyloid A 4	-0.24	0.027	nc	nc	nc	nc
Crp	NM_007768	C-reactive protein, pentraxin-related	0.25	0.044	0.31	0.036	nc	nc
Apcs	BC061125	Serum amyloid P-component	nc	nc	0.71	0.003	-0.86	0.002
