# Supplementary material for: Effect of CAR activation on selected metabolic pathways in normal and hyperlipidemic mouse livers
Source: BMC Genomics. 2009 Aug 19;10:384. doi: 10.1186/1471-2164-10-384 (PMC2739862; doi:10.1186/1471-2164-10-384)
Supplement: Additional file 2 — Systemic effects of TCPOBOP on liver metabolism. Systemic effects of TCPOBOP on liver metabolism. A. Effects of TCPOBOP in conditions of normal diet. B. Effects of 1 week 1% cholesterol diet. C. Effects of TCPOBOP in conditions of high-cholesterol diet. Bold genes, metabolites or processes are up-regulated. [file 1471-2164-10-384-S2.pdf]

Systemic effects of TCPOBOP on liver metabolism

Systemic effects of TCPOBOP on liver metabolism. A. Effects of TCPOBOP in conditions of normal diet. B. Effects of 1 week 1% cholesterol diet. C. Effects of TCPOBOP in conditions of high-cholesterol diet. Bold genes, metabolites or processes are up-regulated.

A

TCPOBOP / normal diet

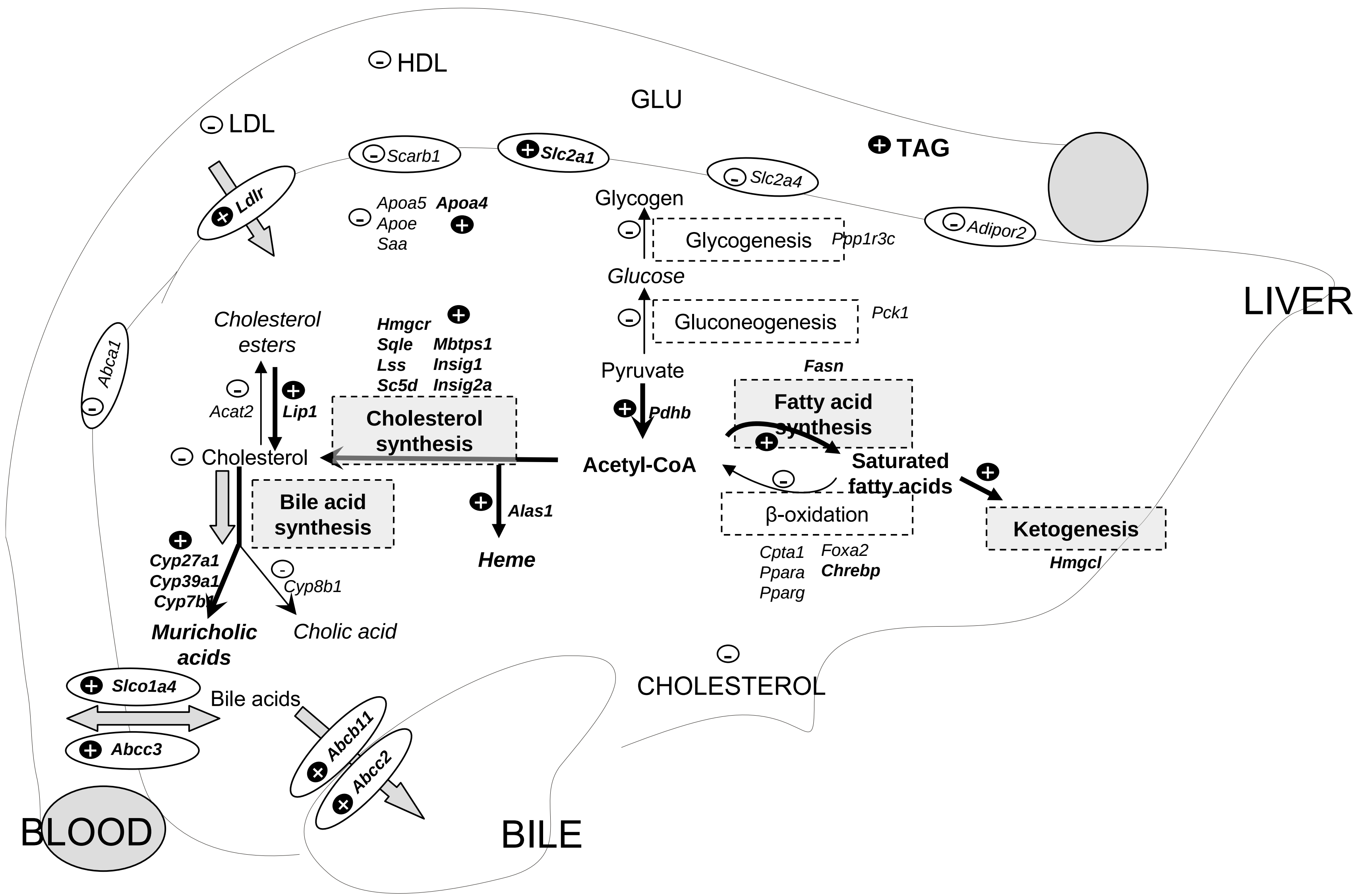

B

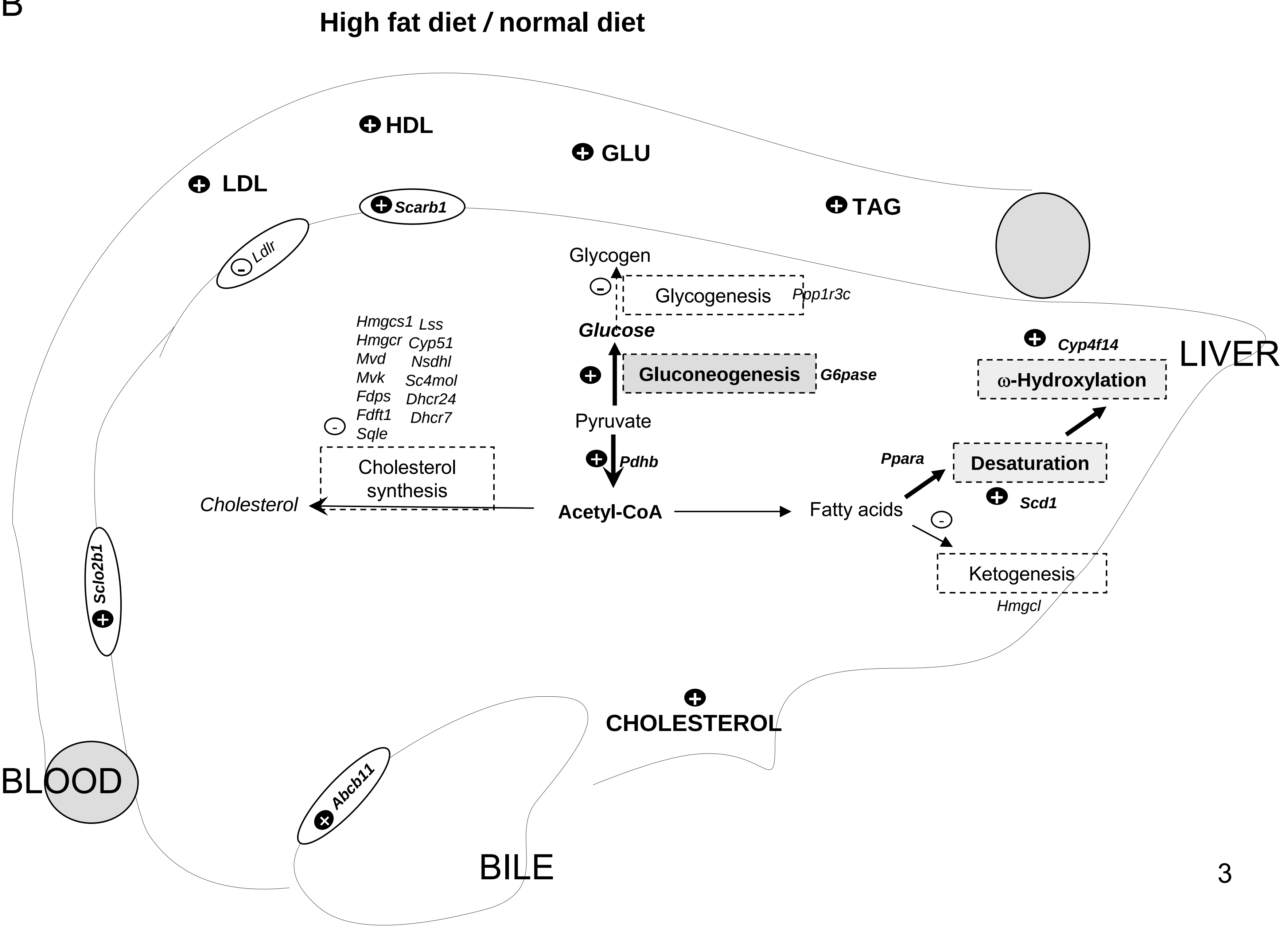
High fat diet / normal diet
HDL
GLU
LDL
TAG
Scarb1
Ldlr
Glycogen
Glycogenesis
Ppp1r3c
Glucose
Gluconeogenesis
G6pase
Pyruvate
Pdhb
Acetyl-CoA
Hmgcs1
Hmgcr
Mvd
Mvk
Fdps
Fdft1
Sqle
Lss
Cyp51
Nsdhl
Sc4mol
Dhcr24
Dhcr7
Cholesterol synthesis
Cholesterol
Fatty acids
Ppara
Desaturation
Scd1
Cyp4f14
ω-Hydroxylation
Ketogenesis
Hmgcl
LIVER
Sclo2b1
CHOLESTEROL
Abcb11
BLOOD
BILE

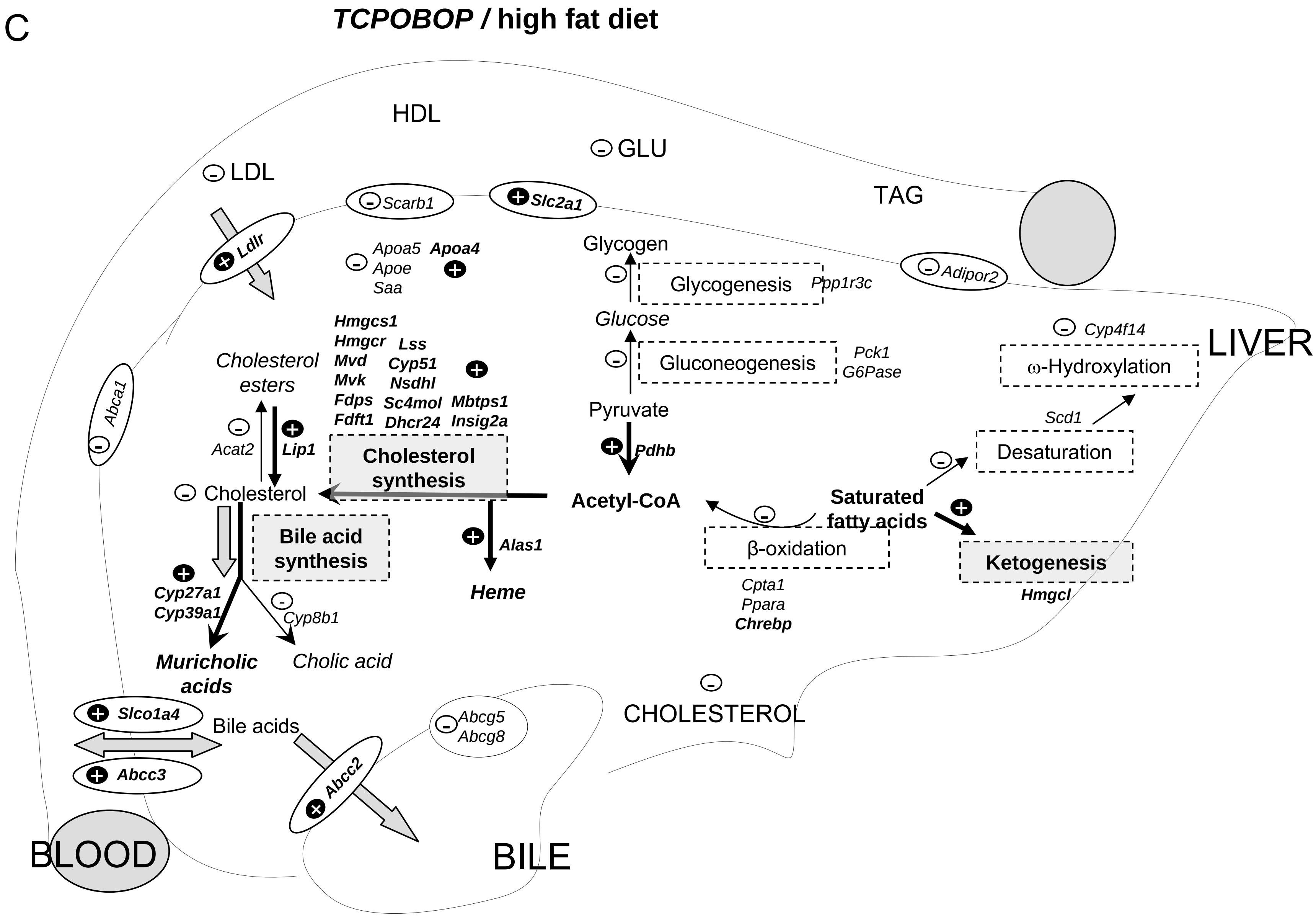
C
TCPOBOP / high fat diet
HDL
GLU
LDL
Scarb1
Slc2a1
TAG
Ldlr
Apoa5
Apoe
Saa
Apoa4
Glycogen
Glycogenesis
Ppp1r3c
Adipor2
Glucose
Gluconeogenesis
Pck1
G6Pase
Cyp4f14
ω-Hydroxylation
LIVER
Hmgcs1
Hmgcr
Mvd
Mvk
Fdps
Fdft1
Lss
Cyp51
Nsdhl
Sc4mol
Dhcr24
Mbtps1
Insig2a
Cholesterol esters
Abca1
Acat2
Lip1
Cholesterol synthesis
Pyruvate
Pdhb
Scd1
Desaturation
Cholesterol
Acetyl-CoA
Saturated fatty acids
Bile acid synthesis
Alas1
Heme
β-oxidation
Ketogenesis
Hmgcl
Cpta1
Ppara
Chrebp
Cyp27a1
Cyp39a1
Cyp8b1
Muricholic acids
Cholic acid
CHOLESTEROL
Slco1a4
Bile acids
Abcg5
Abcg8
Abcc3
Abcc2
BLOOD
BILE
