# Supplementary material for: Effect of CAR activation on selected metabolic pathways in normal and hyperlipidemic mouse livers
Source: BMC Genomics. 2009 Aug 19;10:384. doi: 10.1186/1471-2164-10-384 (PMC2739862; doi:10.1186/1471-2164-10-384)
Supplement: Additional file 3 — Additional data about materials and methods. [file 1471-2164-10-384-S3.pdf]

Additional data about materials and methods

A. Microarray analysis

The Steroltalk microarrays were prepared as follows: 289 clones from the Steroltalk library were amplified using probe-specific primers and AmpliTaq DNA polymerase (Applied Biosystems, Foster City, CA, USA). PCR products were purified using QIAquick 96 PCR Purification Kit (Qiagen, GmBH, Hilden, D) and resuspended at final 100 ng/µL concentration in spotting buffer (50% formamide, 1% CHAPS (3-[(3-cholamidopropyl)-dimethylammonio]-1-propane sulfonate). Probes were spotted on UltraGAPSTM Coated Slides (Corning Inc., Corning, NY, USA) using Biorobotics Microgrid II (Genomic Solutions, Huntington, UK) in a controlled environment (20ºC and 40% humidity). Each probe was spotted in triplicates separately in three blocks. Commercial "spike in controls" Lucidea Universal ScoreCard probes (Amersham Biosciences, GE Healthcare UK limited, Little Chalfont, UK) were also spotted in triplicates in each block.

Samples were prepared as follows: to 20 µg of sample total RNA we added spike in RNA: 250 pg of Firefly Luciferase mRNA (Promega, Madison, WI, USA), and 0.5 µL of either test or reference spike mix from Lucidea Universal ScoreCard kit. We reverse-transcribed mRNA to amino-allyl cDNA using 2.5 µg of Oligo dT (Invitrogen, Carlsbad, CA, USA), 400U of SuperScriptTM III Reverse Transcriptase (Invitrogen, Carlsbad, CA, USA) and 1µL of 10mM amino-allyl dUTP (Sigma, St Louis, MI, USA) according to the manufacturer's protocol. Reaction was stopped after 2 hours by adding 10 µL of 0.5 M EDTA and of 1M NaOH and incubation at 65ºC for 15 min. 10 µL of 1 M HCl was added and cDNA was purified using MinElute PCR Purification Kit (Qiagen GmBH, Hilden, D) according to manufacturer's protocol with exception of using a phosphate buffer (5mM KPO_4 pH 8.5 in 80% ethanol) for washing step and MilliQ water for elution. Purified amino-allyl cDNA was dried and resuspended in 4.5 µL of 0.2 M Na_2CO_3 (pH 9.0) and 4.5 µL of cyanin-3 or cyanin-5 dye in DMSO (Amersham Biosciences, GE Healthcare UK limited, Little Chalfont, UK). Labeling reaction was incubated at room temperature for two hours. Next we added 35 µL of 0.1 M Na acetate (pH 5.2) and purified reaction using MinElute PCR Purification Kit (Qiagen GmBH,

Hilden, D) according to manufacturer's protocol. Labeled cDNA was eluted in water and 1 μL was used for analyses using ND-1000 spectrophotometer (Nanodrop Technologies, Wilmington, Delaware, USA). All test samples were labeled by cianin-3 and the reference samples were labeled cianin-5. No dye-swap experiments were made. Prior hybridization each test sample was mixed with reference sample, hybridization buffer was added (final concentration was 3xSSC, 0.2% SDS) and samples were denatured. Using LifterSlip cover glasses (Erie Scientific Company, Portsmouth, NH, USA) samples were hybridized for 16h at 65ºC in a water bath using humidified hybridization chambers (HybChambers, GeneMachines, San Carlos, CA, USA). Prior hybridizations Steroltalk slides were pre-hybridized for 1 hour at 42ºC in 5xSSC, 0.1%SDS and 1%BSA, then washed at room temperature for 5 min in 2xSSC and 0.1%SDS, 3 min in 0.2xSSC, 2 min in MilliQ water, and dried by centrifugation. After hybridization slides were washed as described before and scanned using Tecan LS200 scanner (Tecan Group Ltd., Maennedorf, CH). Images were analyzed using Array-Pro Analyzer 4.5 (Media Cybernetics, Bethesda, MD, USA). Normalization was done in Orange. As described before data was filtered to exclude spots of low quality and normalized using LOWESS fit to "spike in" control RNAs according to their average intensity (Firefly luciferase and Lucidea Universal ScoreCard). Genes with significant signal in at least 50% of the arrays compared were used.

B. Real-time polymerase chain reaction (RT-PCR) analyses

One microgram of total RNA from individual animals pretreated with DNAse I (Sigma, St Louis, MI, USA) was reverse-transcribed using SuperScriptTM III Reverse Transcriptase (Invitrogen, Carlsbad, CA, USA) and random primers (Promega, Madison, WI, USA) according to manufacturer's protocol. Analyses were done using Platinum® SYBR® Green qPCR SuperMix-UDG (Invitrogen, Carlsbad, CA, USA) and performed on ABI PRISM 7900 HT (PE Applied Biosystems, Foster City, CA, USA) according to manufacturer's protocols in studies of diet effect on TCPOBOP activation. In knock-out experiment analyses were done using LightCycler® 480 SYBR Green I Master and performed on LightCycler® 480 (Roche Diagnostics GmbH, Mannheim, Germany). In both studies internal control was 18S rRNA. Relative transcript levels were calculated

using the comparative Ct (cycle threshold) method and –ΔΔCt values were used for statistical analyses. Student t-test and a probability of type I error α=0.05 was used to determine statistical significance in SPSS 14.0 (SPSS Inc., Chicago, Illinois, USA).

Table 1: Primer sequences used in quantitive RT-PCR analyses.

Gene	Sense primer (5'-3')	Antisense primer (5'-3')
Hmgcr[1]	cttgtggaatgccttgtgattg	agccgaagcagcacatgat
Cyp51	acgctgcctggctattgc	ttgatctctcgatgggctctatc
Cyp8b1[2]	aaggctggcttcctgagctt	aacagctcatcggcctcatc
Cyp7a1[3]	cagggagatgctctgtgttca	aggcatacatcccttccgtga
Cyp3a11[4]	agaacttctccttccagccttgta	gagggagactcatgctccagtta
Cyp2b10[4]	caatgtttagtggaggaactgcg	cactggaagaggaacgtggg
Insig1[5]	tcacagtgactgagcttcagca	tcatcttcatcacacccaggac
Insig2a[5]	ccctcaatgaatgtactgaaggatt	tgtgaagtgaagcagaccaatgt
Insig2b[5]	ccgggcagagctcaggat	gaagcagaccaatgtttcaatgg
Srebp2[1]	gcgttctggagaccatgga	acaaagttgctctgaaaacaaatca
18S[6]	cgccgctagaggtgaaattc	ttggcaaatgctttcgctc
Sqle	tcaaccccagtccagttctc	gactccttcaggtgctcagg

[1]Yang J, Goldstein JL, Hammer RE, Moon YA, Brown MS and Horton JD (2001) Decreased lipid synthesis in livers of mice with disrupted Site-1 protease gene. Proc Natl Acad Sci U S A, 98, 13607-13612; [2]Kok T, Hulzebos CV, Wolters H, Havinga R, Agellon LB, Stellaard F, Shan B, Schwarz M and Kuipers F (2003) Enterohepatic circulation of bile salts in farnesoid X receptor-deficient mice: efficient intestinal bile salt absorption in the absence of ileal bile acid-binding protein. J Biol Chem, 278, 41930-41937; [3]Plosch T, Kok T, Bloks VW, Smit MJ, Havinga R, Chimini G, Groen AK, Kupires F (2002) Increased Hepatobiliary and Fecal Cholesterol Excretion upon Activation of the Liver X Receptor Is Independent of ABCA1. J Biol Chem, 277, 37, 33870-33877; [4]Gnerre C, Schuster GU, Roth A, Handschin C, Johansson L, Looser R, Parini P, Podvinec M, Robertsson K, Gustafsson JA and Meyer UA (2005) LXR deficiency and cholesterol feeding affect the expression and phenobarbital-mediated induction of cytochromes P450 in mouse liver. J Lipid Res, 46, 1633-42; [5] Yabe D, Komuro R, Liang G, Goldstein JL and Brown MS (2003) Liver-specific mRNA for Insig-2 down-regulated by insulin: implications for fatty acid synthesis. Proc Natl Acad Sci U S A, 100, 3155-60; [6]Gutala RV and Reddy PH (2004) The use of real-time PCR analysis in a gene expression study of Alzheimer's disease post-mortem brains. J Neurosci Methods, 132, 101-7.

C. Liver sterol analyses

Liver tissue was weighted, cut into small pieces and placed in a glass vial containing 15 ml Folch solution (chloroform/methanol, 2:1) and 20 µL of 50 mg/ml BHT (butyl hydroxyl toluene). Under inert gas (argon) vials were sealed and left for two days. Approximately 20 mg of liver was used for free lathosterol analyses, 3 mg for total cholesterol and 50 mg for free 7-□-hydroxy-4-cholestene-3-one (C4). Appropriate volume of Folch extract was dried under nitrogen gas and appropriate amount of internal standard and water was added. For free lathosterol and free C4 analyses 100 ng d4-lathosterol or 50 ng d4-C4 in 10 µL MeOH and 10 µL of bidistilled water was added, and evaporated under stream of N2. For total cholesterol 10 µL of bidistilled water, 2 µg d6-cholesterol in 10 µL MeOH, 0.2 mL 33% KOH/water, 1 mL of EtOH and a small magnet was added. The samples were stirring for 1 h at room temperature and evaporated under stream of nitrogen gas. The samples were analyzed by GC-MS with use of an adequate standard curve on a Hewlett Packard HP 5890 Series II Plus Gas chromatograph equipped with a HP 7673A automatic injector and connected to a HP 5972 mass selective detector.

D. Immunoblot analyses of nuclear Srebp2

Approximately 100 mg of frozen tissue was homogenized in 2 mL of homogenization buffer (20mM Tris-HCl at pH 7.4, 2 mM MgCl2, 0.25 M sucrose, 10mM Na-EDTA, 10 mM Na-EGTA,) supplemented with protease inhibitors: 10 mM DTT, 1 mM PMSF, 0.5 mM Pefabloc, 10 µg/mL leupeptin, 5 µg/mL pepstatin A, 10 µg/mL aprotinin, and Complete Mini Protease Inhibitor Cocktail EDTA-free tablets (Roche Molecular Diagnostics GmBH, Mannheim, D). The homogenate was centrifuged at 1000 x g for 5 minutes at 4ºC. Pelet was washed with homogenization buffer and resuspended in 20 mM HEPES-NaOH at pH 7.6, 2.5% V/V glycerol, 0.42 M NaCl, 1.5 mM MgCl2, 1 mM Na-EDTA, and 1 mM Na-EGTA supplemented with Complete Mini Protease Inhibitor Cocktail EDTA-free tablets (Roche Molecular Diagnostics GmBH, Mannheim, D) by continuous mixing for 1 hour at 4ºC. Then we centrifuged the extract at 20.000 x g for 1 hour at 4ºC. Supernatant was transferred to a fresh tube and stored at -80ºC. Protein

concentrations were determined by BSA standard curve using Bio-Rad Protein Assay (Bio-Rad Laboratories Inc., Hercules, CA, USA) and ND-1000 spectrophotometer (Nanodrop Technologies, Wilmington, Delaware, USA) according to manufacturer's protocol. 13 µg of nuclear proteins were mixed with 4x NuPage LDS Sample Buffer (Invitrogen, Carlsbad, CA, USA), denatured and loaded to SDS-PAGE gels NuPAGE® Novex 4-12% Bis-Tris Gels and run in XCellTM Surelock Mini-Cell and NuPAGE® MES SDS Running Buffer (Invitrogen, Carlsbad, CA, USA) according to manufacturer's protocol. Proteins were transferred to PVDF membrane HybondTM-P (Amersham Biosciences, GE Healthcare UK limited, Little Chalfont, UK) using XCell IITM Blot Module and NuPAGE® Transfer Buffer (Invitrogen, Carlsbad, CA, USA) according to manufacturer's protocol. Membrane was incubated in blocking buffer for 1 hour at room temperature, then 1 hour at room temperature in dilution of monoclonal antibodies (2µg/mL mouse anti-Srebp2, BD Biosciences San Jose, CA, USA or 1:100 anti-actin, Sigma, St. Louis, MI, USA). Bound antibodies were visualized with peroxidase-conjugated goat anti-rabbit or anti-mouse IgG (1:2000, Amersham Biosciences, GE Healthcare UK limited, Little Chalfont, UK) and using Supersignal West Pico Chemiluminiscence kit (Pierce Biotechnology, Rockford, IL, USA). Immunoblots of actin were used as loading control.
